# Non-Contact Roughness Measurement in Sub-Micron Range by Considering Depolarization Effects

**DOI:** 10.3390/s19102215

**Published:** 2019-05-14

**Authors:** Franziska Pöller, Félix Salazar Bloise, Martin Jakobi, Shengjia Wang, Jie Dong, Alexander W. Koch

**Affiliations:** 1 Institute for Measurement Systems and Sensor Technology, Technical University of Munich, Theresienstrasse 90, 80333 Munich, Germany; m.jakobi@tum.de (M.J.); shengjia.wang@tum.de (S.W.); jie.dong@tum.de (J.D.); a.w.koch@tum.de (A.W.K.); 2 ETSI Minas y Energía, Universidad Politécnica de Madrid, Calle de Ríos Rosas 21, 28003 Madrid, Spain; felixjose.salazar@upm.es

**Keywords:** roughness measurement, non-contact method, depolarization, contrast

## Abstract

The characteristics of a surface, particularly the roughness, play an important role in different fields of the industry and have to be considered to ensure quality standards. Currently, there are numerous sophisticated methods for measuring surface roughness but plenty of them cause long-term damage because they are in contact with the sample. This article presents a non-contact method to accurately determine small surface roughnesses resulting from the consideration of the depolarization effects caused by the rough surface. This technique can be applied as an extension in various roughness measurements and improves the approach of *Chandley’s* technique, which does not take into account the depolarization of the light scattered by the sample. The experimental setup and the measurements are easy to perform. The essential component is a quarter wave plate, which is incorporated into a Michelson interferometer. With the resulting two different contrasts and the recorded intensities of the sample and the reference mirror, the surface roughness can be estimated straightforwardly. This article details the theoretical approach, followed by the experimental results and the corresponding uncertainties. The experimental results are compared with *Chandley’s* method. In order to have reference roughness values of the samples, measurements with a stylus profilometer and with a confocal microscope are performed and compared.

## 1. Introduction

In many areas it is important to have detailed knowledge of the structure of a surface. In various fields of application, surface roughness plays an especially important role: in medicine, surface roughness influences the compatibility of implants with the human body [1] and the bacterial plaque retention on dental materials [2]; in pharmaceutics, the chemical composition of tablets can be analyzed [3]; in the field of aerospace engineering, surface roughness has an impact on flight dynamics [4]; in the fabrication of machined workpieces, surface roughness effects their quality [5].

In general, there are two different approaches to measure surface roughness, namely contact and non-contact methods. One of the most important methods for measuring roughnesses is the mechanical profilometer. This apparatus has proven to be very useful, but its displacement along many parallel lines on the surface under study is time-consuming. Furthermore, the contact techniques are not always suitable for determining roughness, for instance, with soft surfaces where the tiny tip may damage the sample. Nowadays, there is an increasing number of sensitive surfaces in the industry and therefore the demand for non-contact methods is rising.

Moreover, the diverse technologies to determine the roughness of a surface can be grouped in three categories—namely profiling methods (1), topography methods (2) and area-integrating methods (3).

Profiling devices, such as stylus instruments and optical profilometers, evaluate the roughness by way of a surface profile, which is a certain specimen line $z\left(x\right)$. These contacting methods provide reliable results but the stylus width is not always narrow enough to resolve the surface structures [6,7]. Topography methods generate an outcome which is portrayed as a topological picture $z\left(x,y\right)$ and could consist of many parallel lines. This type of measurement includes, for instance, focus variation microscopy [8,9], coherence interferometry such as white light interferometry [10,11,12], fringe protection [13] and laser or white light confocal microscopy [14,15,16]. With these methods, it is possible to obtain a complete distribution of surface roughness values, which is time-consuming. Area-integrating methods, such as light scattering methods [17] and speckle correlation [18], measure surface roughness, relying on the micro structure of a certain region of the surface. This technique characterizes the roughness as a statistical value, for example, it gives the mean value of the surface height distribution, which reflects the roughness.

Among the different techniques, optical methods represent an important scope. Since the reflected light contains information about the surface, the roughness can be estimated by analyzing the optical wave. *Chandley* developed a non-contact method, which is a mixture of type (2) and (3), to measure the roughness using an interference microscope that takes advantage of the relation between the surface roughness and the fringe visibility in an interferogram [19].

However, in this method, it is supposed that the light scattered by the rough surface does not change its polarization state. In this article, we present a non-contact technique for determining surface roughness, in which the polarization of the reflected radiation may change and which also represents a combination of the type (2) and (3) methods. We consider depolarization effects produced by the rough surface. Compared with the method of *Chandley*, this procedure promises higher accuracy of the estimated surface roughness for small roughnesses by introducing a quarter wave plate (QWP) in a part of the reference beam, thus giving different contrasts in the interferometric fringe pattern. Generally, a fringe pattern is generated by using a plane and tilted reference wave and the scattered wave from the surface [20]. By means of this setup, two different contrast values on the same picture are obtained; each of them is dependent on the surface roughness [21] and on the variation of the degree of the polarization of the light.

Surface control is one of the most important parameters of any surface and it is a deciding factor when particularly high surface quality requirements need to be met. The proposed non-contact method is intended to be an improvement of *Chandley’s* technique [19]. In this context, it can be used to improve the accuracy of roughness measurements in the sub-micron ranges of samples whose depolarization effects cannot be neglected. In general, this improved technique can be used in the field of coatings [22] with numerous industrial applications. For instance, this method may be viable for determining the roughness of the surface finish of optical components based on silicon and germanium (IR optics), optical glasses in lens manufacturing [23], or in polymer components (CR-39, PMMA) used as substrates for depositing coatings [24], indium tin oxide (ITO) films [25], coatings for tribological performances [26], and some TiO_2_ layers for medical applications [27], among others.

The rest of the article is structured as follows: Firstly, the theoretical approach of the Depolarization-Based Roughness Measurement (DBRM) method is introduced; secondly, the experimental results are presented and discussed; later, the propagation of the expanded uncertainty of the measurement results is analyzed.

## 2. Materials and Methods

### 2.1. Theoretical Approach

**Figure 1:**
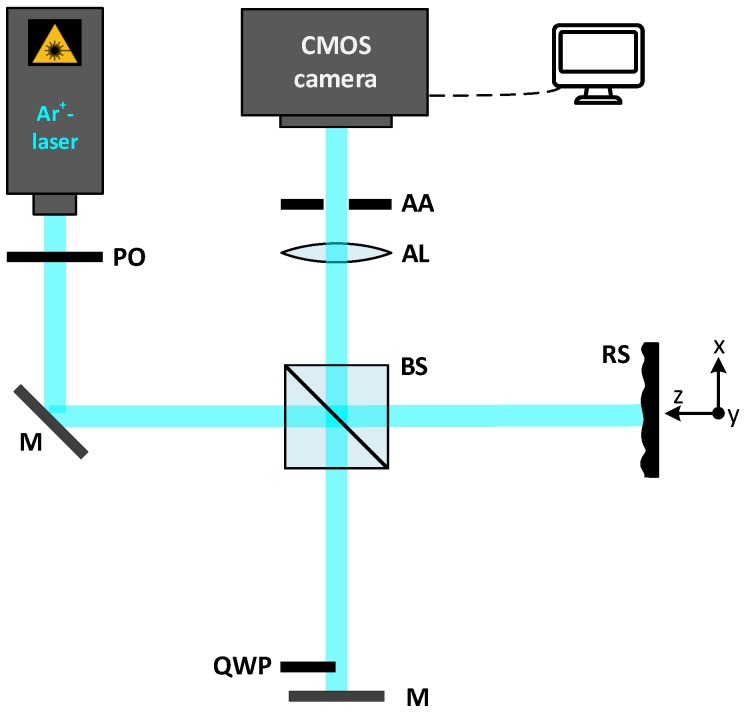
Experimental setup. PO: polarizer; M: plane mirror; BS: (non-polarizing) beam splitter; RS: rough surface; quarter wave platet (QWP): quarter wave plate; AA: adjustable aperture; AL: achromatic lens; CMOS: complementary metal-oxide semiconductor.

The proposed system is based on a Michelson interferometer modified with a quarter wave plate in a part of the reference beam, as shown in Figure 1.

A polarizer is located at the output of the laser source to guarantee a linearly polarized beam. Let ${\vec{E}}_{0}=\left(0,E_{y0}\right)$ be the field which impinges the rough surface after passing through the non-polarizing beam splitter (BS). This beam strikes the sample and the scattered light changes its polarization state as a consequence of the roughness of the sample, thus modifying the electric field components to ${\vec{E}}_{s}=\left(E_{xs},E_{ys}\right)$ where the subscript *s* stands for the sample. The other part of the beam is directed onto a slightly tilted plane mirror (M), in which a quarter wave plate has been placed on its left side. As a result, the electric field of the light reflected by the mirror (M) in the reference path can be divided into two parts, namely *A* and *B*. The first (part *A*) corresponds to the right part of the mirror and the left region (part B) is where the QWP is inserted. Due to the inclusion of the wave plate, the reflected electric field at the output of the QWP varies its plane of polarization; then, we have two plane reference waves, linearly polarized, with their respective electric fields perpendicularly disposed, that is, ${\vec{E}}_{m}^{A}=\left(0,E_{ym}\right)$ (part *A*) and ${\vec{E}}_{m}^{B}=\left(E_{xm},0\right)$ (part *B*) where the subscript *m* refers to the mirror. The experimental effect of having two reference waves with their electric fields perpendicular to each other is to obtain a different contrast of the interference pattern at each part *A* and *B*. Taking into account the field components of the light reflected by the sample and the tilted reference mirror, the averaged intensities registered on the camera, $\langle I_{A}\rangle$ and $\langle I_{B}\rangle$, belonging to part *A* and part *B*, respectively, are

(1) $$\begin{array}{cc} \langle I_{A}\rangle & =\langle I_{A}^{y}\rangle +\langle I_{A}^{x}\rangle =I_{ys}+I_{ym}+2\sqrt{I_{ys}I_{ym}}\exp\left(-k\sigma_{z}^{2}\right)\cos\left(2kf\left(x,y\right)\right)+I_{xs} \end{array}$$

(2) $$\begin{array}{cc} \langle I_{B}\rangle & =\langle I_{B}^{x}\rangle +\langle I_{B}^{y}\rangle =I_{xs}+I_{xm}+2\sqrt{I_{xs}I_{xm}}\exp\left(-k\sigma_{z}^{2}\right)\cos\left(2kf\left(x,y\right)\right)+I_{ys}, \end{array}$$

where $k=\frac{2\pi }{\lambda }$ denotes the wave number with the wavelength of the laser light λ. Here, $\sigma_{z}$ is the standard deviation of the height on a rough surface and $f\left(x,y\right)$ is the function that indicates the shape chosen for the reference. In the present case, we choose the function corresponding to a tilt around the *x*- or *y*-axis, thus the interference pattern is formed by parallel straight lines [19]. Furthermore, the surface must be flat, which in our case is always guaranteed. For the further derivation, we suppose the well-known contrast of the fringes $C_{M}$ as

(3) $$C_{M}=\frac{I_{max}-I_{min}}{I_{max}+I_{min}},$$

where $I_{max}$ denotes the maximum intensity and $I_{min}$ the minimum intensity [28]. By inserting the intensity values from Equations (1) and (2) into Equation (3), we obtain the following expressions for the two contrasts $C_{A}$ (part *A*) and $C_{B}$ (part *B*)

(4) $$\begin{array}{cc} C_{A} & =\frac{2\sqrt{I_{ys}I_{ym}}\exp\left(-k\sigma_{z}^{2}\right)}{I_{ys}+I_{ym}+I_{xs}}\approx \frac{2\sqrt{I_{ys}I_{ym}}\exp\left(-k\sigma_{z}^{2}\right)}{I_{ys}+I_{xs}+I_{0}} \end{array}$$

(5) $$\begin{array}{cc} C_{B} & =\frac{2\sqrt{I_{xs}I_{xm}}\exp\left(-k\sigma_{z}^{2}\right)}{I_{xs}+I_{xm}+I_{ys}}\approx \frac{2\sqrt{I_{xs}I_{xm}}\exp\left(-k\sigma_{z}^{2}\right)}{I_{xs}+I_{ys}+I_{0}}, \end{array}$$

in which we assume that the intensities of the reflected light on parts *A* and *B* are nearly the same, namely:

(6) $$I_{xm}\approx I_{ym}=I_{0}.$$

Experimentally, we know the value of the sum $I_{ys}+I_{xs}$ and Equation (6) applies. The contrasts $C_{A}$ and $C_{B}$ (see Equations (4) and (5)) can also be extracted from the experiments. The problem is to identify the values of $I_{ys}$ and $I_{xs}$ themselves that are required to determine the roughness of the surface. If we had no depolarization of the light $I_{xs}=I_{0}\approx 0$, one equation for the contrast would be sufficient to obtain a value for the roughness. In our case, the sample is depolarizing, so we would require two experiments. Thanks to the QWP, we have the divided, and therefore the required, two pieces of information on the camera, thus we have to carry out the experiment only once. To obtain the values of $I_{ys}$ and $I_{xs}$, we need the following equation

(7) $$I_{xs}+I_{ys}=I_{s},$$

where $I_{s}$ represents the total intensity of the specimen on the CMOS camera. By inserting Equations (4), (5) and (7) into each other, we have:

(8) $$I_{ys}=\frac{I_{s}{\left(\frac{C_{B}}{C_{A}}\right)}^{2}}{\left(1+{\left(\frac{C_{A}}{C_{B}}\right)}^{2}\right)}=\frac{I_{s}C_{A}^{2}}{\left(C_{A}^{2}+C_{B}^{2}\right)}\;\mathrm{and}\;I_{xs}=\frac{I_{s}{\left(\frac{C_{A}}{C_{B}}\right)}^{2}}{\left(1+{\left(\frac{C_{A}}{C_{B}}\right)}^{2}\right)}=\frac{I_{s}C_{B}^{2}}{\left(C_{A}^{2}+C_{B}^{2}\right)}.$$

After rearranging Equation (4) and solving the equation for $\exp\left(-k\sigma_{z}^{2}\right)$, we obtain the following expression:

(9) $$\frac{\left(I_{xs}+I_{ys}+I_{0}\right)\sqrt{C_{A}^{2}+C_{B}^{2}}}{2\sqrt{I_{0}I_{s}}}=\exp\left(-k\sigma_{z}^{2}\right).$$

The left side of the term is known from the experiments, thus the roughness equals:

(10) $$\sigma_{z}=\frac{\lambda }{4\pi }\sqrt{\ln\left(\frac{4I_{0}I_{s}}{{\left(I_{s}+I_{0}\right)}^{2}\left(C_{A}^{2}+C_{B}^{2}\right)}\right)}.$$

It would make no difference if we did the same calculation by first rearranging Equation (5) instead of Equation (4); we would obtain the same roughness (see Equation (10)). As per definition, the standard deviation of the height on a rough surface $\sigma_{z}$ equals the root mean square (RMS) roughness $S_{q}$ [18,29], we can also write Equation (10) as:

(11) $$S_{q}=\sigma_{z}.$$

Before describing the measurement setup and the procedure in detail, we need to make some theoretical assumptions. The standard deviation of the height on a rough surface $\sigma_{z}$ must be Gaussian distributed. As can be seen from Equations (1) and (2), we assume a Gaussian distribution $\exp\left(-k\sigma_{z}^{2}\right)$, otherwise we would have received other formulas. This is the basis of our theory, as well as that for *Chandley’s* method.

### 2.2. Experimental Setup

For the experiments, the modified Michelson setup depicted in Figure 1 that is used to explain the theory in Section 2.1, is employed. The collimated and, by an optical fiber, extenuated laser beam passes through a polarizer (PO) in order to control the initial polarization conditions. The used light source is an Ar^+^-laser head (LEXEL 3500) with laser lines, which range from 457.9 nm to 514.5 nm. The laser is employed in single-line operation. To verify the feasibility of our method, the wavelength of $\lambda_{\mathrm{Ar}+}=488.0\;\mathrm{nm}$ for the measurements is chosen.

A 50:50 non-polarizing beam splitter (BS) divides the beam into an object path and a reference path. The laser light, which is linearly polarized in the *y*-direction (see Figure 1), is guided to a tilted plane mirror (M), in which a quarter wave plate is partly positioned and adjusted with its optical axis to $45{}^{\circ }$ on one of its halves (part *B*) [30,31]. Thus, we have two different polarization states of the reference beam after reflecting on the mirror, which allows the division of the information on parts *A* and *B* on the complementary metal oxide semiconductor (CMOS) camera. Physically, this division is like having two Michelson interferometers in the same setup, each with a different reference laser beam from the polarization viewpoint. At the same time, the light is scattered by the rough surface (RS), thus producing two speckled interference fringe patterns (*A* and *B*) on the CMOS camera. This camera (Photonfocus) has a resolution of 1312×1082 pixels, a pixel size of $8\mu m^{2}$ and a resolution of 12 bit.

To obtain an image with a sharp resolution on the camera, an adjustable aperture (AA) and an achromatic lens (AL), which has a focal length of f=80 mm, is used. The magnification is set at a value of $M=\frac{b}{f}=\frac{12\;\mathrm{cm}}{80\;\mathrm{mm}}=1.5$ where *b* is the distance from the achromatic lens (AL) to the CMOS camera.

### 2.3. Measurement Process and Data Evaluation

**Figure 2:**
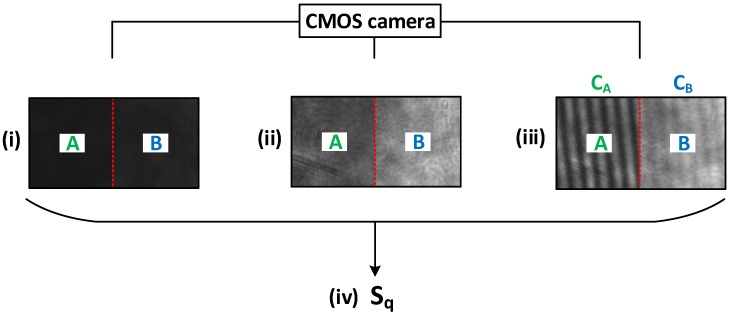
Measurement procedure and data evaluation. *A*: part *A* without the QWP, *B*: part *B* with the introduced QWP; $C_{A}$: contrast of part *A*; $C_{B}$: contrast of part *B*. (**i**) Measurement 1: Record of the reflected light of the rough surface; (**ii**) Measurement 2: Record of the reflected light of the plane mirror; (**iii**) Measurement 3: Record of the reflected light with the information from both areas to determine $C_{A}$ and $C_{B}$; (**iv**) Calculation of the RMS roughness $S_{q}$ (see Equation (11)) (Images from the measurement of a surface with the RMS roughness $S_{q}=31\;\mathrm{nm}$ at a wavelength of $\lambda_{\mathrm{Ar}+}=488.0\;\mathrm{nm}$).

In this section, the measurement process is described in detail following all the steps demonstrated in the theory. As we can see from Equation (10), we need to experimentally determine $I_{s}$, $I_{0}$ and the contrasts $C_{A}$ and $C_{B}$. This measurement process and the subsequent data evaluation are schematically depicted in Figure 2.

The procedure consists of three measurements. Firstly, the CMOS camera records the reflected light of the rough sample (see Figure 2i). The intensity values of part *A* and part *B* represent the intensity of the rough surface on the CMOS camera $I_{s}$ (see Equation (7)). The second measurement is the recording of the reflected light of the plane mirror (see Figure 2ii), which is synonymous with the intensity on the plane mirror $I_{0}$, which we assume to be as in Equation (6). Then, the camera records the third image, which is necessary for the calculation of the contrasts (see Figure 2iii). We determine the contrasts by means of the contrast $C_{M}$ (see Equation (3)). If we apply Equations (10) and (11), we obtain the desired RMS roughness $S_{q}$ of the sample (see Figure 2iv), in which the depolarization effects have been taken into account.

In the measurement procedure, the intensity reflected from part *B* differs from the intensity of part *A* of the plane mirror. The QWP inserted in part *B* between the plane mirror and the beam splitter absorbs some radiation (about 8% back and forth), resulting in an inaccurate measurement of the $I_{0}$ intensity of part *B*. In order to avoid this problem, another glass with the same absorbtion characteristics, but not birefringent, was located in part *A*; thus, the intensity $I_{0}$ at both parts is the same (see Equation (6)).

The presented method takes *Chandley’s* technique as a particular case. In fact, by setting $C_{B}$ to zero we obtain the values of the roughness without considering depolarization effects. This is equivalent to applying *Chandley’s* formula

(12) $$\sigma_{c}={\left[-\frac{2}{k^{2}u^{2}}\ln\left(\langle v_{0}\rangle \right)\right]}^{1/2},$$

where the subscript *c* stands for the abbreviation of Chandley, *k* is again the wave number and *u* is the geometrical factor [32], which in our case is set to 2 because the system acts in reflection. The quantity $\langle v_{0}\rangle$ denotes the normalized ensemble averaged tilt fringe visibility [19], which can be written as:

(13) $$\langle v_{0}\rangle =\exp\left(-\frac{{\left(ku\sigma_{c}\right)}^{2}}{2}\right),$$

where $\sigma_{c}$ is also equal to the RMS roughness $S_{q}$ (see Equation (11)).

**Figure 3:**
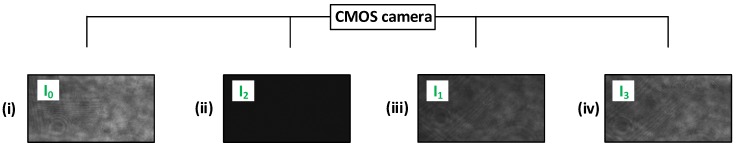
Measurement procedure to obtain the intensities for the calculation of the Stokes parameters $S_{0}$, $S_{1}$, $S_{2}$, and $S_{3}$. (**i**) Measurement 1: Record of the intensity $I_{0}$; PO: $0{}^{\circ }$; (**ii**) Measurement 2: Record of the intensity $I_{2}$; PO: $90{}^{\circ }$; (**iii**) Measurement 3: Record of the intensity $I_{1}$; PO: $45{}^{\circ }$; (**iv**) Measurement 4: Record of the intensity $I_{3}$; PO: $45{}^{\circ }$ and QWP: $45{}^{\circ }$ (Images from the measurement of a surface with the RMS roughness $S_{q}=31\;\mathrm{nm}$ at a wavelength of $\lambda_{\mathrm{Ar}+}=488.0\;\mathrm{nm}$).

To verify the assumption that the rough surface depolarizes the impinging light, we also determine the degree of polarization (DOP) for every sample. To some extent, we can relate the DOP to the roughness of the surface. So, in principle, the higher the surface roughness, the higher the depolarization. For calculating the degree of polarization, we use the well-known expression

(14) $$DOP=\frac{\sqrt{S_{1}^{2}+S_{2}^{2}+S_{3}^{2}}}{S_{0}}\leq 1,$$

in which $S_{0}$, $S_{1}$, $S_{2}$, and $S_{3}$ are the Stokes parameters. We need only four different intensity parameters to characterize the polarization state of the light. Therefore, four different values of intensity must be measured by using a polarizer and an analyzer. The intensity $I_{0}$ is observed when the polarizer is set to $0{}^{\circ }$ (see Figure 3i); to get the intensity $I_{1}$, we set the polarizer to $45{}^{\circ }$ (see Figure 3iii) and for the intensity $I_{2}$, the polarizer must have a position of $90{}^{\circ }$ (see Figure 3ii). For the recording of the intensity $I_{3}$, we rotate the polarizer to $45{}^{\circ }$ and introduce the QWP, which stands at $45{}^{\circ }$ in the reference beam (see Figure 3iv). The values of the Stokes parameters as a function of the intensities are the following [33,34]:

(15) $$\begin{array}{cc} S_{0} & =I_{0}+I_{2} \end{array}$$

(16) $$\begin{array}{cc} S_{1} & =I_{0}-I_{2} \end{array}$$

(17) $$\begin{array}{cc} S_{2} & =2\;\cdot \;I_{1}-I_{0}-I_{2} \end{array}$$

(18) $$\begin{array}{cc} S_{3} & =2\;\cdot \;I_{3}-I_{0}-I_{2}. \end{array}$$

## 3. Results

To verify the theoretical approach of the DBRM method, some measurements are executed. This section explains the experimental results, which are compared to the results generated by *Chandley’s* method. In order to justify the results of the RMS roughness $S_{q}$ being more accurate if we take the depolarization effect of the surface into account, we also obtain the degree of polarization (DOP). The experiments are carried out with the laser line of $\lambda_{\mathrm{Ar}+}=488.0\;\mathrm{nm}$. This wavelength is chosen here because it has a high intensity and lies in the middle of the full range of the Ar^+^-laser head.

As mentioned before, the presented method and *Chandley’s* technique are valid for a Gaussian distributed surface.

### 3.1. Experimental Results

We measure five samples with distinct surface roughnesses to confirm the validity of our measurement method and to examine its limits. For each sample, we calculate the degree of polarization (DOP) to emphasize the importance of taking the depolarization of rough surfaces into account to obtain more accurate results. We determine the DOP according to Equation (14). Moreover, the experimental results are compared to the method of *Chandley*, the stylus profilometer and the confocal microscope. It is important to say that the only aim of this comparison is to establish a reference value of the roughness for each sample, thus inferring the improvement of the DBRM technique with respect to *Chandley’s* method, and its limits of validity, but not to analyze whether the proposed experimental procedure is better or worse than the profilometer or the confocal microscope.

Because, in an actual case, the results of the $S_{q}$ (according to Equations (10) and (11)) may slightly differ from one part of the surface to another, for each specimen three measurements are executed in distinct regions for nine different sections per experiment. Afterwards, the calculated RMS roughnesses $S_{q}$ are averaged. The same applies when we obtain the experimental results with *Chandley’s* technique. Furthermore, for the calculation of the DOP, three different sections in the measurement are evaluated and then averaged. With the stylus profilometer, the roughness $R_{q}$ for twenty profiles pointing in different directions on the surface is determined for each sample and then averaged. This provides a suitable mean value and allows us to identify the one-dimensional parameter $R_{q}$ and the two-dimensional or area-related roughness parameter $S_{q}$. All of the experimental results are depicted in Table 1.

Our proposed method gives the RMS roughness $S_{q}$ of a surface and is a consequence of the hypothesis of the theory exposed, namely that the scattering surface has a first order probability density function of heights, which is Gaussian with standard deviation $\sigma_{z}$ (see Equations (1) and (2)). Furthermore, it is one of the usual parameters employed in other optical procedures as, for instance, speckle correlation methods [18,29,35]. Thus, we confine ourselves to the roughness parameter $S_{q}$.

Generally, surface roughness measurements are difficult to compare and to assess when using different methods. The stylus profilometer and our method and/or *Chandley’s* technique are different measurement procedures. Because the stylus profiler (SURFCOM FLEX 50A) belongs to the profiling techniques (the radius of the diamond tip is 2 μm and the stylus has a measuring force of 0.75 mN) and the confocal microscope (SENSOFAR) is an optical method which is assigned to the topography procedures as well as to the area-integrating techniques, the values obtained may differ. Besides, small defects on the surface structure itself can lead to an interpretation error. It is also possible that the surfaces are not exactly Gaussian distributed and then the theoretical assumptions are not exactly fulfilled [35,36,37,38,39]. However, even though the results cannot match exactly, it is important to have these values as a reference in its order of magnitude to make a comparison between *Chandley’s* technique and the proposed method possible.

Another problem that may exist is that the section considered to obtain the RMS roughness $S_{q}$ differs from each other. The area of the stylus profiling consists only of single lines, and in our method $S_{q}$, differs from a quadratic section and also the single $S_{q}$ differs from different quadratic areas.

Moreover, the technique is much more sensitive, since the total area considered is smaller than that of the stylus profiler. In addition, many other parameters have a strong influence on surface roughness, such as wear and friction [40].

A closer look at the experimental results in Table 1 reveals that the values obtained by means of the DBRM method are closer to these determined by the stylus profiler and the confocal microscope. Thus, the roughness parameter $S_{q}$ for small surface roughnesses calculated with the proposed technique are better than those calculated using *Chandley’s* method. It is also noticeable that the DOP decreases with a higher surface roughness, as supposed. These reliable results are an indicator for considering the depolarization effects of rough surfaces. Taking into consideration the results of the stylus profiler and the confocal microscope as reference values, the theory is only compatible for small roughnesses up to a value of about $R_{q}=37\;\mathrm{nm}$ (sample 3), in contrast with the reference methods, which seem to be more appropriate beyond this interval. From this value onwards, the values of both procedures begin to vary because a rougher surface produces a more speckled fringe interference pattern. The values are not acceptable and no longer valid, then reaching its limit of validity. As a consequence, the fringes needed to obtain the value for the contrasts (see Equations (4) and (5)) are no longer very visible, making the application of the method not viable. In fact, noisy signals due to speckles are an inherent problem of all speckle interferometric techniques for measuring system properties/characteristics by using intensity fringes from an interferogram. So, by increasing the roughness of the sample surface, a speckle pattern is superimposed to the fringes (signal), which leads to a blurred intensity picture, preventing the measurement to account. This does not apply to the stylus profilometer and the confocal microscope. They perform the measurements without noisy signals. In contrast to these and other methods, which are accurate in estimating a high surface roughness [32,41,42], the presented technique is especially reliable for small roughnesses.

### 3.2. Expanded Uncertainty of the Method

To analyze the accuracy of the proposed procedure, we will first examine the combined standard uncertainty $u_{c}^{2}\left(S_{q}\right)$ of the method. Supposing that the variables in Equation (10) are not correlated (covariances are zero), we apply the following equation, which is commonly used for propagating uncertainties [43,44]

(19) $$u_{c}^{2}\left(S_{q}\right)=\sum_{i=1}^{N}{\left(\frac{\partial f}{\partial x_{i}}\right)}^{2}u^{2}\left(x_{i}\right),$$

where *f* represents the RMS roughness $S_{q}$ of the surface heights ($S_{q}=\sigma_{z}$, see Equation (11)), and $u\left(x_{i}\right)$ is the standard uncertainty for each input quantity in Equation (10) (λ, $I_{0}$, $I_{s}$, $C_{A}$, and $C_{B}$), which must be obtained through the evaluation of Type A and Type B analysis, i.e., $u\left(x_{i}\right)=\sqrt{{\left(u_{A}\left(x_{i}\right)\right)}^{2}+{\left(u_{B}\left(x_{i}\right)\right)}^{2}}$ [43]. Once this calculation is performed, the expanded measurement uncertainty may be easily determined by employing the equality

(20) $$U\left(S_{q}\right)=ku_{c}\left(S_{q}\right),$$

*k* being a coverage factor related with a defined confidence interval. All results shown in Table 1 are computed by choosing k=2, which gives a confidence interval of about 95%.

## 4. Discussion

In conclusion, the presented method shows promising results for small roughnesses. The experimental setup is easy to implement and the measurements and the estimation of surface roughness can be carried out straightforwardly. For small roughnesses, the method is more accurate than *Chandley’s* method according to the conducted comparison, which is depicted in Table 1. Also, the results of the surface roughnesses measured by the confocal microscope and the stylus profilometer confirm the outcomes of the DBRM method for small roughnesses. Furthermore, the suggested method is non-destructive. In addition, the uncertainties sustain the outcome of the article that the introduced method is a suitable method for determining the roughness of a surface in the sub-micron range and it can be used as an extension in various roughness measurement techniques to improve the estimated values. The uncertainties in Table 1, which are small at a roughness value of up to $R_{q}=37\;\mathrm{nm}$, support the conclusion that the DBRM method can be used for processes where small surface roughnesses are required, such as in optic manufacturing for the nano-finishing of surfaces of optical components, in the field of coatings and for some applications in medicine.

Though, the key element, and simultaneously the most error-prone component in the system, is the quarter wave plate. It has to be adjusted carefully to guarantee the correct polarization and thus acceptable measurement results. Last but not least, the random uncertainties of the experimental results in Table 1 could be further reduced. Furthermore, the resolution of the experimental setup could be improved and the accuracy of the technique can be further enhanced by an intensity-stable light source, or a quarter wave plate with a high quality is required.

It is also conceivable that surface roughness can be evaluated point by point to produce two-dimensional data in a map, which allows a clear allocation of the location of the roughnesses and possible damages or other defects of the surface.

This article assumes a Gaussian distribution of the standard deviation of the height on the rough surface $\sigma_{z}$, which is the basis of the theoretical approach. In order to extend the method, other distributions can also be assumed as the foundation for theoretical considerations, such as a Laplace distribution or a K-distribution [45,46].

## Figures and Tables

**Table 1:** Experimental results for five samples measured by different methods, namely the stylus profiler, the confocal microscope, the DBRM technique and *Chandley’s* method, respectively. The uncertainties of the techniques and the degrees of polarization (DOP) are also given.

	Sample 1	Sample 2	Sample 3	Sample 4	Sample 5
stylus profiler ($R_{q}$)	28±2nm	31±2nm	37±2nm	45±2nm	116±4nm
confocal microscope ($S_{q}$)	26±1nm	28±1nm	52±1nm	49±1nm	116±1nm
DBRM method ($S_{q}$)	27±1nm	31±1nm	32±2nm	28±3nm	31±4nm
method by *Chandley* ($S_{q}$)	40±2nm	41±2nm	44±2nm	44±2nm	49±1nm
DOP	0.825	0.785	0.709	0.669	0.588
